# Neurotropic Viruses, Astrocytes, and COVID-19

**DOI:** 10.3389/fncel.2021.662578

**Published:** 2021-04-09

**Authors:** Petra Tavčar, Maja Potokar, Marko Kolenc, Miša Korva, Tatjana Avšič-Županc, Robert Zorec, Jernej Jorgačevski

**Affiliations:** ^1^Laboratory of Neuroendocrinology–Molecular Cell Physiology, Institute of Pathophysiology, Faculty of Medicine, University of Ljubljana, Ljubljana, Slovenia; ^2^Celica Biomedical, Ljubljana, Slovenia; ^3^Institute of Microbiology and Immunology, Faculty of Medicine, University of Ljubljana, Ljubljana, Slovenia

**Keywords:** SARS-CoV-2, COVID-19, flavivirus, neurotropic virus, neuroinfection, astrocyte

## Abstract

At the end of 2019, the severe acute respiratory syndrome coronavirus 2 (SARS-CoV-2) was discovered in China, causing a new coronavirus disease, termed COVID-19 by the WHO on February 11, 2020. At the time of this paper (January 31, 2021), more than 100 million cases have been recorded, which have claimed over 2 million lives worldwide. The most important clinical presentation of COVID-19 is severe pneumonia; however, many patients present various neurological symptoms, ranging from loss of olfaction, nausea, dizziness, and headache to encephalopathy and stroke, with a high prevalence of inflammatory central nervous system (CNS) syndromes. SARS-CoV-2 may also target the respiratory center in the brainstem and cause silent hypoxemia. However, the neurotropic mechanism(s) by which SARS-CoV-2 affects the CNS remain(s) unclear. In this paper, we first address the involvement of astrocytes in COVID-19 and then elucidate the present knowledge on SARS-CoV-2 as a neurotropic virus as well as several other neurotropic flaviviruses (with a particular emphasis on the West Nile virus, tick-borne encephalitis virus, and Zika virus) to highlight the neurotropic mechanisms that target astroglial cells in the CNS. These key homeostasis-providing cells in the CNS exhibit many functions that act as a favorable milieu for virus replication and possibly a favorable environment for SARS-CoV-2 as well. The role of astrocytes in COVID-19 pathology, related to aging and neurodegenerative disorders, and environmental factors, is discussed. Understanding these mechanisms is key to better understanding the pathophysiology of COVID-19 and for developing new strategies to mitigate the neurotropic manifestations of COVID-19.

## Introduction

**FIGURE 1:**
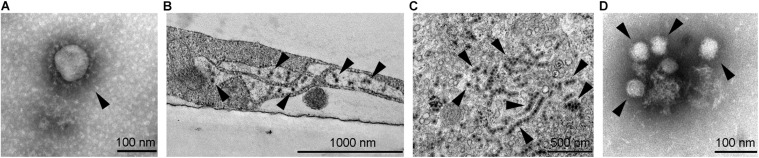
Transmission electron micrographs of selected neurotropic viruses. **(A)** SARS-CoV-2 (marked by arrowhead) isolated on Vero E6 cells, ultracentrifuged onto formvar coated with carbon stabilized grids and negatively stained by using 2% phosphotungstic acid. **(B)** West Nile viruses (marked by arrowheads) in an infected SK-N-SH cell; the image shows an Epon embedded ultrathin section contrasted with uranyl acetate and lead citrate. **(C)** Zika virus (see arrowheads, strain Uganda 976) in an infected Vero E6 cell; the image shows an Epon embedded ultrathin section contrasted with uranyl acetate and lead citrate. Strings of viruses represent virus localization within endoplasmic reticulum. **(D)** Tick-borne encephalitis virus (marked by arrowheads) isolated on Vero E6 cells, ultracentrifuged onto formvar coated with carbon stabilized grids and negatively stained using 2% phosphotungstic acid. Electron micrographs were obtained by transmission electron microscope (JEM-1400 Plus, JEOL, Tokyo, Japan) at 120 kV.

Human coronaviruses (CoVs) were first identified in the mid-1960s and were named for the crown-like spikes on their surface (Figure 1A). The newly identified severe acute respiratory syndrome coronavirus 2 (SARS-CoV-2) belongs to β-CoVs, which also include SARS-CoV Middle East respiratory syndrome (MERS-CoV), and human coronavirus OC43 and HKU1 (HCoV-OC43 and HCoV-HKU1, respectively). The primary target cells for SARS-CoV-2 are the epithelial cells of the respiratory and gastrointestinal tract that contain angiotensin-converting enzyme 2 (ACE2), which is utilized by the virus to enter the cell. However, the penetration of this viral agent into the organism is most likely not limited only to these tissues (Li et al., 2020). Indeed, there is evidence that SARS-CoV-2 affects the central nervous system (CNS) through which it also contributes to the pathophysiology of COVID-19 (Steardo et al., 2020).

Recent post-mortem high-resolution magnetic resonance microscopy imaging of the brain and histopathological examination of the olfactory bulb and brain stem of COVID-19 patients revealed perivascular-activated microglia, macrophage infiltrates, and hypertrophic astrocytes. Activated microglia were found adjacent to neurons, suggestive of neuronophagia, in the olfactory bulb, substantia nigra, dorsal motor nucleus of the vagal nerve, and the pre-Bötzinger complex in the medulla, which is involved in the generation of spontaneous rhythmic breathing (Lee et al., 2020). In this latter brain structure, astrocytes have been considered to mediate the detection of the environmental changes related to the control of breathing (Gourine et al., 2010). Currently, the presence of SARS-CoV-2 in the CNS is still debated. However, upon infection and other forms of brain damage, neuroglial cells become reactive, the hallmark of which is hypertrophy, which represents the most classic neuropathological scenario of ongoing neuroinflammation (Pekny and Pekna, 2016), as observed in the afore-mentioned post-mortem study (Lee et al., 2020). Astrocytes play a myriad of functions that support neurons. As such, they are considered the key homeostasis-providing cells in the CNS (Verkhratsky et al., 2019b). Moreover, astrocytes provide a favorable environment to support replication of viruses, since they exhibit aerobic glycolysis. This special adaptation of metabolism exists in rapidly dividing cells and in cells undergoing plastic morphological changes, despite the presence of adequate levels of oxygen, a phenomenon known as “the Warburg effect” (Vander Heiden et al., 2009), typically present in cancer cells. While this form of metabolism is not very efficient in producing ATP, it is the biosynthetic intermediates of this metabolism, that provide an essential advantage for cells in developing and growing tissues (Tech and Gershon, 2015) also supporting the replication of viruses (Zorec et al., 2019). Therefore, neuroinfection may contribute to the pathophysiology of COVID-19 through impaired astroglial function.

## Neurotropic Viruses Affect the Functions of Astrocytes and Neurons

A vast variety of viruses from different families are capable of invading the CNS in which they infect different cell types. For a long time, neurons were the primary focus of studies investigating neuroinfections; however, in the past decade, other cell types in the CNS have gained attention. Astrocytes are among the most important cells in the course of CNS infections due to several reasons: (i) they are abundant and the first cell type to be infected after viruses cross the blood–brain barrier (BBB); (ii) they produce and release high amounts of progeny virus, as demonstrated for certain flaviviruses; and (iii) they release immunomodulating molecules that affect the survival of neurons (Potokar et al., 2019). Here, we focus on neurotropic viruses from the *Coronaviridae* and *Flaviviridae* families. These two families comprise viruses that represent important human pathogens, some of which have caused recent and ongoing epidemics. Besides the well-known neurotropic pathogens, such as the tick-borne encephalitis virus (TBEV) and West Nile virus (WNV), also viruses that were previously not typically associated with neurotropism, such as Zika virus (ZIKV) and the new SARS-CoV-2 (Figure 1) are associated with severe and possibly lethal neurological symptoms.

## Coronaviruses (Family *Coronaviridae*)

Coronaviruses are enveloped, positive-strand RNA viruses that were considered negligible human pathogens, causing the “common cold” in otherwise healthy people and only causing more severe symptoms in children, the elderly, and immunocompromised individuals (Bohmwald et al., 2018; Paules et al., 2020). CoVs are respiratory viruses, along with the respiratory syncytial virus, influenza virus, and human metapneumovirus, that are also responsible for CNS human neuropathologies, such as febrile seizures, loss of consciousness, convulsion, ataxia, status epilepticus, encephalitis, myelitis, and neuritis (Bohmwald et al., 2018). Before the global SARS-CoV-2 epidemic, several other CoVs had already been detected in the CNS of human patients, such as HCoV-229E, SARS-CoV, and HCoV-OC43 (Bohmwald et al., 2018). However, none of these viruses caused such extensive and severe human pathologies on a global scale as SARS-CoV-2 has (though, the lack of modern technologies likely resulted in underestimated magnitudes of virus outbreaks back in the day).

### SARS-CoV-2 as a Neurotropic Virus

Infection with SARS-CoV-2 may result in neurological and neuropsychiatric symptoms; more than 35% of COVID-19 patients develop neurological symptoms (Niazkar et al., 2020). These are more common in severe manifestations of the disease and can vary between individuals (Montalvan et al., 2020; Niazkar et al., 2020). Neuropathological conditions observed in COVID-19 patients include seizures, encephalopathy, encephalitis, meningitis, headaches, anosmia, ageusia, nausea, decreased alertness, dizziness, impaired consciousness, reduced cognition, delirium, depression, acute cerebrovascular disease, acute disseminated encephalomyelitis, acute necrotizing hemorrhagic encephalopathy, and acute Guillain-Barré syndrome [thoroughly reviewed in Montalvan et al. (2020), Najjar et al. (2020), and Niazkar et al. (2020); see also Jarrahi et al., 2020; Tremblay et al., 2020].

Since the first descriptions of neurological manifestations related to SARS-CoV-2 infection, many efforts have been devoted to elucidating the possible cellular mechanisms affected by SARS-CoV-2 that lead to abnormal functioning of the central and peripheral nervous systems. Neuroinflammation, which commonly accompanies CNS damage, can be induced directly by viral invasion into the CNS or indirectly by systemic hyperinflammation. The latter is caused by excessive activation of the innate immune system, which results in the release of pro-inflammatory mediators following SARS-CoV-2 infection, and is an important hallmark of COVID-19 (Najjar et al., 2020). In both scenarios, astrocytes are likely important players, as they are known target cells for infection with other neurotropic viruses, such as flaviviruses (reviewed below) and human immunodeficiency virus type 1 (HIV-1) (Chauhan et al., 2014). This is predominantly due to two characteristics of astrocytes. First, astrocytes are very abundant in the CNS, especially along the possible entry routes of viruses into the CNS. Second, astrocytes can act as immune modulators in the CNS, contributing to immune responses and releasing cytokines, chemokines, and growth factors following different insults (Dong and Benveniste, 2001; Colombo and Farina, 2016; Soung and Klein, 2020).

### Entry of SARS-CoV-2 Into the CNS

In 2000 two independent publications reported the discovery of a novel human zinc metalloprotease that shared substantial homology (∼40%) to ACE and was therefore termed ACE2 (Donoghue et al., 2000) and ACEH (Tipnis et al., 2000), respectively. ACE and ACE2 are vital proteases that regulate the renin–angiotensin system (RAS) and hence control cardiovascular and renal functions by maintaining homeostasis of blood pressure (Iwai and Horiuchi, 2009). Therefore, it is not surprising that ACE2 is predominantly expressed in the heart, kidney, gastrointestinal tract, lung, and testes (Donoghue et al., 2000; Tipnis et al., 2000); though, the expression of ACE2 was confirmed also in the brain (Komatsu et al., 2002). In the brain, ACE2 activity was shown to affect not only central cardiovascular regulation, but also modified levels of various other substrates, such as apelin, neurotensin, kinin, and opioid peptides (Alenina and Bader, 2019). These pleiotropic actions of ACE2 in the brain were associated with several distinct processes ranging from stress response and anxiety to neurogenesis and cognition (Alenina and Bader, 2019; and references within).

It is well accepted that the entry of SARS-CoV-2 into a host cell is mediated by ACE2, which functions as an entry receptor (Hoffmann et al., 2020). The interaction between viral spike (S) glycoproteins and ACE2 facilitates SARS-CoV-2 attachment onto the surface of the target cell; this is followed by virus entry that is likely mediated by clathrin-mediated endocytosis (Bayati et al., 2020; Petersen et al., 2020). According to a transcriptome database analysis, ACE2 is expressed in the majority of brain regions (e.g., the amygdala, cortex, frontal cortex, substantia nigra, and hippocampus) but mostly in low quantities (Chen et al., 2020). In the human brainstem, the highest ACE2 expression levels were found in the pons and medulla oblongata, which contain the medullary respiratory centers of the brain (Lukiw et al., 2020). Higher ACE2 levels are also present in the choroid plexus and paraventricular nucleus of the thalamus (Chen et al., 2020). Analysis of human and mouse brain showed that ACE2 is expressed predominantly in neurons but also in non-neuronal cells, including astrocytes, oligodendrocytes, endothelial cells, and pericytes. Interestingly, ACE2 RNA levels in endothelial cells and pericytes were high in the mouse brain but low in the human brain (Chen et al., 2020). It has been demonstrated that SARS-CoV infection, and even recombinant S protein on its own, can downregulate ACE2 expression (Kuba et al., 2005; Glowacka et al., 2010). Considering the aforementioned multifaceted features of ACE2 activity in the brain, one may assume that its downregulation may result in various deleterious consequences, such as deterioration of cognition (Wang et al., 2016). Several reports suggest that COVID-19 can present with neurological deficits, including memory impairment (Garg et al., 2020; Lu et al., 2020), which is also supported by a COVID-19 clinical update showing that individuals who recovered from COVID-19 perform worse on cognitive tests in multiple domains (Hampshire et al., 2020; a preprint). Nevertheless, further studies are required to assess the involvement of altered ACE2 activity in COVID-19 neurological manifestations.

The presence of ACE2 makes cells susceptible to SARS-CoV-2 infection; however, in order to infect the CNS, the virus must first invade the brain, which is protected from the external environment by the BBB and blood-cerebrospinal fluid-barrier (BCB) (Pellegrini et al., 2020). There are multiple pathways of viral entry into the CNS, including infection of sensory nerve endings, motor neurons at neuromuscular junctions, olfactory epithelium, olfactory neurons, and endothelial cells of the BBB as well as invasion of infected leukocytes from the circulatory system (i.e., the Trojan horse mechanism) (Koyuncu et al., 2013). In the case of SARS-CoV-2, the current evidence points to two most plausible mechanisms of brain invasion: (i) entry into the CNS via the olfactory pathway by axonal transport along infected olfactory nerves and then dissemination through *trans*-synaptic transmission to other brain areas (Montalvan et al., 2020; Yachou et al., 2020) or (ii) the hematogenous pathway via either infected blood cells (usually leukocytes) from the circulatory system or infected endothelial cells of the BBB. The hematogenous pathway may also involve infection of epithelial cells of the choroid plexus, the building blocks of the BCB (Montalvan et al., 2020; Murta et al., 2020; Vargas et al., 2020; Yachou et al., 2020). The latter mechanism seems to be the preferred pathway of SARS-CoV-2 neuroinvasion, as different research groups have reported a high susceptibility of choroid plexus epithelial cells to SARS-CoV-2 infection (Jacob et al., 2020; Pellegrini et al., 2020) and have observed changes in BCB integrity following SARS-CoV-2 infection (Pellegrini et al., 2020). Moreover, SARS-CoV-2 RNA has been detected not only in brain autopsy samples (Puelles et al., 2020) but also in the cerebrospinal fluid of COVID-19 patients (Moriguchi et al., 2020; Virhammar et al., 2020). The presence of the virus in post-mortem human brain tissue was also confirmed at the protein level by immunological detection (Song et al., 2020). Of note, virus entry is most likely facilitated by changes in the permeability and integrity of the BBB during systemic hyperinflammation (Alquisiras-Burgos et al., 2020; Murta et al., 2020; Najjar et al., 2020). Another intriguing mechanism via which SARS-CoV-2 may spread is through the vagus nerve from infected lungs (Jarrahi et al., 2020).

### Organoids as Models for the Study of SARS-CoV-2 Infection

At the level of *in vitro* biological samples, human brain organoids, derived from human induced pluripotent stem cells, are considered a valuable tool for investigating SARS-CoV-2 neurotropism (Bullen et al., 2020; Jacob et al., 2020; Pellegrini et al., 2020; Ramani et al., 2020; Song et al., 2020; Zhang B.-Z. et al., 2020). Findings suggest that epithelial cells of choroid plexus are the primary target of SARS-CoV-2 infection in the CNS, as choroid plexus organoids showed the highest infection rate and productive viral replication (Jacob et al., 2020; Pellegrini et al., 2020). This is consistent with the finding that the choroid plexus brain region is one of the hotspots of ACE2 expression in the CNS (Chen et al., 2020; Jacob et al., 2020) and thus more prone to SARS-CoV-2 infection. Cell junction remodeling and increased inflammatory responses were observed in choroid plexus epithelial cells following SARS-CoV-2 infection (Jacob et al., 2020). In agreement with these results, Pellegrini et al. (2020) also presented evidence that SARS-CoV-2 infection of choroid plexus organoids results in altered BCB integrity. They proposed that BCB disintegration could facilitate the entry of viruses as well as immune cells and cytokines into the cerebrospinal fluid and brain tissue, possibly inducing neuroinflammation (Pellegrini et al., 2020).

Besides epithelial cells of choroid plexus, neurons, astrocytes, and neural progenitor cells in brain organoids are also susceptible to SARS-CoV-2 infection (Bullen et al., 2020; Jacob et al., 2020; Ramani et al., 2020; Song et al., 2020; Zhang B.-Z. et al., 2020), although the infection rates of the respective cell types remain under debate. Song et al. (2020) reported that the majority of infected cells in brain organoids correspond to mature neurons, while multipotential neural stem cells are prone to infection to a lesser extent. Immunological detection in brain organoid preparations revealed the presence of SARS-CoV-2 membrane (M) protein mostly in neuronal soma and in some cases also in neurites (Bullen et al., 2020). Electron microscopy further demonstrated the presence of SARS-CoV-2 in neurons, as viral particles were observed budding from the endoplasmic reticulum, indicating that the virus can replicate in this cell type (Song et al., 2020). Conversely, other authors report sparse infection of neurons and astrocytes (Jacob et al., 2020). Pellegrini et al. (2020) even reported that neurons and glial cells in their organoid preparations do not get infected with SARS-CoV-2 pseudovirions or with live virus, except in cases of high live virus concentrations. Interestingly, it seems that also microglial cells are not susceptible to SARS-CoV-2 infection (Jacob et al., 2020). Similar to the debate regarding the ability of SARS-CoV-2 to infect different cell types in the CNS, also viral replication in the CNS still remains a controversial theme. Several reports that measured viral RNA and/or released viral particles indicate that SARS-CoV-2 replication is successful in brain organoids (Bullen et al., 2020; Song et al., 2020; Zhang B.-Z. et al., 2020), while others suggest that viral replication and spread are not efficient in the brain (Jacob et al., 2020; Ramani et al., 2020). These opposing results may arise from differences between organoid models and virus concentrations and passages that were used in the respective studies. We must also point out that although human brain organoids represent an important and much needed tool for *in vitro* studies of SARS-CoV-2 infection, these models have a simplified structure, resembling the developing fetal brain, and lack mature cells (especially astrocytes and microglial cells), vasculature, and a BBB (Jacob et al., 2020; Ramani et al., 2020). In the future, the neurotropic properties of SARS-CoV-2 must be further explored on cells and tissues obtained from human brain samples. For example, ACE2 expression and the presence of viral spike (S) protein were already confirmed in cortical neurons in post-mortem brain samples from COVID-19 patients (Song et al., 2020).

### Astrocytes and SARS-CoV-2 Infection

To date, the majority of studies have focused on exploring the effects of SARS-CoV-2 on neurons; however, there is a need to study astrocytes as well. Alterations in astrocytic metabolism by neurotropic viruses most likely impact neuronal functioning, as it is well accepted that astrocytes play a crucial role in supporting neurons with energy intermediates and defense mechanisms (Sofroniew and Vinters, 2010; Kreft et al., 2012; Zorec et al., 2019).

Viral infections, including those by SARS-CoV-2, and other CNS insults are known to trigger reactive astrogliosis (Lee et al., 2020), an evolutionarily conserved process that includes alterations in the gene expression, biochemistry, and morphology of astrocytes (Murta et al., 2020). Depending on the insult, astrocytes can shift to a destructive pro-inflammatory phenotype, increasing the release of cytokines, chemokines, and neurotoxic factors and thus promoting CNS damage (Colombo and Farina, 2016; Murta et al., 2020). Reactive astrocytes can also become facultative antigen presenting cells (Bozic et al., 2020) and attract immune cells, e.g., leukocytes, to the lesion site, further contributing to immune cell infiltration and thus neuroinflammation (Colombo and Farina, 2016). Importantly, CNS damage in COVID-19 patients is indicated by elevated GFAP plasma levels (Kanberg et al., 2020); this suggests that astrocyte activation may be involved in SARS-CoV-2 neuropathogenesis. A pro-inflammatory state can be induced not only by a direct viral infection but also by the mere presence of cytokines and other inflammatory mediators, evading from the blood circulation (Murta et al., 2020). Indeed, many argue that the devastating neurological damage caused by SARS-CoV-2 is not a consequence of direct infection of neural cells but rather a result of systemic inflammation. This phenomenon is typical for severe COVID-19 pathogenesis, which eventually leads to BBB alterations and neuroinflammation (Najjar et al., 2020; Tremblay et al., 2020). Nevertheless, positive viral staining of post-mortem brain tissues of COVID-19 patients does not coincide with leukocyte or lymphocyte infiltration (Song et al., 2020). This finding suggests that SARS-CoV-2-related neurological complications may be a direct result of the neurovirulent properties of the virus.

Another aspect that must be elucidated in future studies is whether astrocyte activation, which possibly occurs following SARS-CoV-2 infection, impacts BBB integrity. Astrocytes are structurally and functionally important for BBB formation and maintenance, as they enwrap brain capillaries by specialized end-feet processes and provide soluble signaling factors necessary for tight junction formation (Cabezas et al., 2014; Colombo and Farina, 2016). Astrocytic infection and/or activation may therefore lead to the disruption of this barrier, leading to an invasion of toxic molecules and immune cells into the brain. Of note, reactive astrocytes, neuroinflammation and/or BBB integrity alterations have been implicated in many neurodegenerative and neuropsychiatric disorders (for example Alzheimer’s disease, Parkinson’s disease, multiple sclerosis, epilepsy, cerebral ischemia, stroke, neurotrauma, and bipolar disorder) and neuroinfections (Cabezas et al., 2014; Verkhratsky et al., 2014; Patel and Frey, 2015; Pekny et al., 2016). For example, HIV invades the brain by hematogenous pathway. Although HIV infection of astrocytes is sparse and mostly unproductive in terms of viral replication, it affects BBB physiology and it has been linked to neurological disorders (Eugenin et al., 2011). The main mechanism involved in astrocyte-dependent BBB disruption includes toxic signal spreading from infected astrocytes to surrounding non-infected cells involving gap-junctions, endothelial cell apoptosis and changes in astrocyte end-feet formation, localization, and signaling (Eugenin et al., 2011). Similarly, ZIKV infection of astrocytes results in a loss of the BBB integrity, which is accompanied with massive blood cell infiltration (especially CD8^+^ T cells) into the CNS (Jurado et al., 2018). Additionally, infection with Japanese encephalitis virus (JEV) induces a strong pro-inflammatory response [signified by elevated levels of interleukin-6 (IL-6), CCL5, and CXCL10] in endothelial cells of the BBB and in astrocytes, leading to increased BBB permeability and possibly further facilitating virus entry into the brain (Patabendige et al., 2018). The situation may also be reverse, i.e., the disruption of the BBB caused by pro-inflammatory mediators from circulating blood or viral infection of brain endothelial cells could lead to astroglial and microglial activation (Alquisiras-Burgos et al., 2020; Chen and Li, 2021). In line with this scenario, SARS-CoV-2 was identified in the endothelial cells in the frontal lobe of COVID-19 patients (Paniz-Mondolfi et al., 2020), whereas the spike (S) protein of SARS-CoV-2 has been found to promote a pro-inflammatory response in brain endothelial cells *in vitro* (Buzhdygan et al., 2020; a preprint). It has also been demonstrated that arboviruses and CoVs, which also possess neuroinvasive properties, compromise BBB integrity by replicating in brain microvascular endothelial cells (Paniz-Mondolfi et al., 2020). Based on the knowledge gained from other neurotropic viruses, we can hypothesize that impaired BBB structure and function would facilitate SARS-CoV-2 entry into the CNS, leading to more severe neurological complications. Similar to other viruses (i.e., influenza virus, HIV and herpes simplex virus type 1) that invade the CNS, and activate inflammatory and immune responses (De Chiara et al., 2012; Zhou et al., 2013; and references within), SARS-CoV-2 infection could itself be an exposome (i.e., all non-genetic exposures of an individual in a lifetime) factor contributing to the risk for developing neurological disorders.

COVID-19 disease manifestation can vary tremendously between infected individuals from asymptomatic cases to severe and life-threatening scenarios, which are much more common in elderly and in people with comorbidities (Butler and Barrientos, 2020; Gao et al., 2021; Hu et al., 2021). The underlying reasons behind high symptom variability in healthy individuals are still sparse; however, they may, in part, depend also on environmental impact, life style and previous disease status (Naughton et al., 2020; Hu et al., 2021). External factors, such as air pollution, high fat diet, consumption of refined carbohydrates, nicotine consumption, and certain drugs, are hypothesized to affect the course of COVID-19 course of disease through the level of ACE2 expression and inflammation in specific tissues (Naughton et al., 2020; Hu et al., 2021; and references within). Diet rich in saturated fats induces chronic inflammation through toll-like receptor pathways and can impair functions of the adaptive immune system (via increased oxidative stress), leading to unsuccessful response to pathogen infections (Butler and Barrientos, 2020).

Moreover, when considering neurological signs and symptoms and the role of astrocytes in this matter, lung-brain and gut-brain axis should be taken into account in SARS-CoV-2 infected individuals. Namely, SARS-CoV-2 lung damage is hypothesized to cause direct or indirect neuronal effects, probably through virus-induced inflammation and oxidative stress (Stevens and Puybasset, 2011; Nuzzo and Picone, 2020). In addition, the gut-brain axis which connects gastrointestinal system with cognitive centers of the brain, is implicated in SARS and MERS infections through gut microbiome. By analogy, also COVID-19 may, to some level, be linked to the enteric microbiota and innate immunity mechanisms (Ahlawat et al., 2020; Chaves Andrade et al., 2020; Shinu et al., 2020). Astrocytes play an integral role in the development of several neurodegenerative disorders, as neuroinflammation is a driving factor of disease severity (Ma et al., 2019; Borsom et al., 2020), although a novel study reveals that a subset of anti-inflammatory astrocytes may limit CNS inflammation relayed by the microbiome (Sanmarco et al., 2021). Therefore, the relationship between astrocytes and specific microbiota in particular pathologic conditions need to be addressed individually in the future.

Astrocyte shape determines memory formation (Zorec et al., 2015) and provides for homeostasis and defense of the CNS. Alterations of astroglial cellular processes, and consequently their interactions with neurons, may be affected by aging- and neurodegeneration-related changes (Verkhratsky et al., 2020). In aging and various pathologies, astrocytes undergo morphofunctional remodeling including atrophy, asthenia and loss of function (Plata et al., 2018; Verkhratsky et al., 2019a, 2020). These changes may deteriorate the course of COVID-19 in patients with Alzheimer’s disease and related dementias (ADRD), epilepsy, neuromyelitis optica spectrum disorder (NMOSD), putting elderly population and those with pre-existing diseases to a higher risk of fatal COVID-19 outcome. For example, patients with ADRD are at higher risk of COVID-19 morbidity and mortality, not only because of behavioral and cognitive problems (Brown et al., 2020), but also because of comorbidities, such as cardiovascular disease, diabetes, Parkinson’s disease, stroke, atherosclerosis and pneumonia; the latter being roughly twice as prevalent in individuals with dementia compared to individuals without this condition (Bauer et al., 2014; Foley et al., 2015). At the cellular level, important contributors to higher morbidity and mortality of SARS-CoV-2 infected patients with neurodegenerative diseases should be sought also in astroglia. Namely, SARS-CoV-2 infection of already altered astrocytes may severely deteriorate the brain conditions. For example, in elder people and those with neurodegenerative disorders, such as depression, ADRD and NMOSD, serum levels of several pro-inflammatory cytokines, including IL-6, are elevated (Ng et al., 2018; Wei et al., 2018). IL-6 plays an important role in cytokine release syndrome (CRS), a systemic inflammatory response characterized by a sharp increase in the level of a large number of pro-inflammatory cytokines, which has been linked to severe COVID-19 cases (Zhang C. et al., 2020). In lungs, binding of SARS-CoV-2 to alveolar epithelial cells activates the innate and adaptive immune systems, resulting in the release of a variety of cytokines, including IL-6 (Zhang C. et al., 2020). In the CNS the physiological function of IL-6 is multifaceted; on one hand IL-6 exerts neurotrophic and on the other hand neuroprotective effects, and can also function as a mediator of inflammation, demyelination, and astrogliosis (Van Wagoner and Benveniste, 1999). The predominant source of IL-6 in the CNS is reactive astrocytes, and while they retain IL-6 levels low in the normal brain, they elevate IL-6 expression during injury, stroke, inflammation, and infection (Zhang C. et al., 2020). The mechanism of release of gliosignaling molecules and cytokines, involving regulated exocytosis (Stanley and Lacy, 2010) may be affected by SARS-Cov-2 proteins, in a similar manner as affected by other neurotropic viruses (Guček et al., 2016). For example, elevated infection-evoked production of multiple cytokines and chemokines, including IL-6 has been detected after infection with flaviviruses in serum and cerebrospinal fluid of patients, brain tissue samples from mice and primary human cortical astrocyte cultures (Palus et al., 2014; Stefanik et al., 2018; Pokorna Formanova et al., 2019). While both neurons and astrocytes are potential sources of pro-inflammatory cytokines in flavivirus-infected brain tissue, astrocytes are the main producers of cytokines/chemokines that stimulate the innate neuronal immune response (Pokorna Formanova et al., 2019). The notion that the immune response initiated to eradicate virus becomes pathological, causing immune-mediated damage to the host, has been documented clinically and experimentally (King et al., 2007).

Further studies are needed to fully understand the role of astrocytes in SARS-CoV-2-mediated CNS damage. The current evidence implies that these cells may be considerably involved in viral replication and spread in the CNS and could play a role in inducing or further promoting neuroinflammation and neurotoxicity after infection.

## Flaviviruses (Family *Flaviviridae*)

Besides CoVs, viruses from other families can also be neurotropic, including flaviviruses. These are transmitted to humans mainly by arthropods, such as mosquitos and ticks, and possibly by other yet unknown vectors (Mandl, 2005). As with CoVs, flavivirus transmission between humans has also been documented (Mlakar et al., 2016; Arora et al., 2017; Counotte et al., 2018). Flaviviruses might enter the CNS by infecting peripheral and olfactory neurons and brain endothelial cells, thus breaching the BBB and BCB (Potokar et al., 2019). The olfactory route, a potential route of entry into the CNS for SARS-CoV-2 (Fodoulian et al., 2020), is also one of the confirmed entry points for flaviviruses, such as the JEV, in primates (Myint et al., 1999). In addition, the presence of several other flaviviruses (e.g., the Langat virus and TBEV) has already been confirmed in the olfactory bulb (Lindqvist et al., 2016, 2018).

### Flavivirus Infection of Astrocytes

**FIGURE 2:**
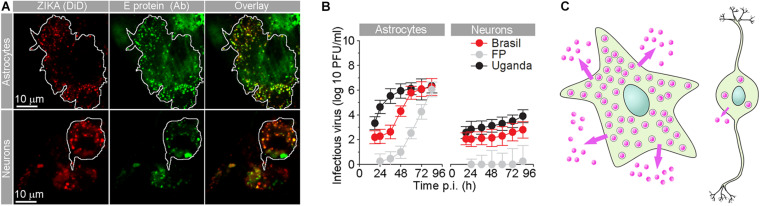
Human fetal astrocytes are more efficiently infected with Zika virus and release more progeny virus than neurons. **(A)** Micrographs show the internalized fluorescently labeled Brazilian Zika virus (ZIKV) strain in astrocytes and neurons. Prior to infection, Zika virus were labeled by lipophilic Vybrant DiD dye [ZIKA (DiD)], and 36 h post-infection (p.i.), cells were immunolabeled with serum from a patient infected with ZIKV (infected in Brazil in 2016). Overlay panels show remarkable co-localization between vesicular structures with fluorescently labeled ZIKV and anti-ZIKV antibodies from the patient’s serum. The cell boundaries of individual astrocytes and neurons are delineated. Note that the number of Zika particles is significantly higher in astrocytes compared to neurons. **(B)** The graphs represent plaque assay measurements of infectious virus particles in the supernatants at different times p.i. of three ZIKV strains [Brazil 2016 (Brazil), French Polynesia 2013 (FP), and Uganda #976 1947 (Uganda)]. The production trend of infectious virus particles in astrocytes is higher than that in neurons for all three strains. In both astrocytes and neurons, infectious ZIKV-FP virus particles exhibited the lowest concentrations. At 12 h p.i., the supernatant did not contain countable plaques of ZIKV-FP viral particles. **(C)** A schematic representation depicting higher production and release of progeny ZIKV virus (pink dots) in astrocytes compared to neurons. Modified from Jorgačevski et al. (2019) with permission.

Several flaviviruses have been recognized as neurotropic and are successful in infecting astrocytes (Potokar et al., 2019). In flavivirus infections, astrocytes stand out due to their high production of the virus in comparison to other cell types in the CNS. In comparison to neurons (Figure 2; Jorgačevski et al., 2019), astrocytes produce orders of magnitude more virus, as demonstrated for ZIKV, TBEV, and WNV (Cheeran et al., 2005; Palus et al., 2014; Potokar et al., 2014; Lindqvist et al., 2016). This is highly relevant for the spread of infection through the CNS, especially because astrocytes are also more resilient to flavivirus infection. Astrocytes provide a potential reservoir for virus retention and production in the CNS; however, despite their massive production of TBEV, ZIKV, and WNV, astrocytes retain higher viability than neurons (Diniz et al., 2006; Palus et al., 2014; Jorgačevski et al., 2019). Murine postnatal and human fetal astrocytes can establish persistent WNV and ZIKV infections *in vitro* that last for weeks, implying astrocyte involvement in chronic or persistent WNV infection in the CNS (Diniz et al., 2006; Potokar et al., 2014; Limonta et al., 2018). Regardless of their resilience, TBEV, ZIKV, and JEV infections trigger substantive changes in human astrocytes, including their stress-induced reactivation, morphological changes of intracellular organelles (e.g., endoplasmic reticulum, Golgi complex, mitochondria, and phagosomes), and altered dynamics (i.e., size and mobility) of virus-laden vesicles due to infection-inflicted redistribution of the cytoskeleton (Mishra et al., 2008; Palus et al., 2014; Potokar et al., 2014, 2019). All these changes enable host cells to support viral replication and spread.

The role of astrocytes in host immune responses is well recognized and two-sided. On the one hand, increased expression and release of pro-inflammatory cytokines from astrocytes was confirmed in cell cultures and patients and contributes to the modulation of host immune responses and flavivirus-induced neurotoxicity. This includes BBB breakdown, which further decreases neuronal viability and consequently exacerbates the course of the disease (Van Marle et al., 2007; Růžek et al., 2011; Bardina and Lim, 2012; Palus et al., 2013, 2014; Chang et al., 2015; Li et al., 2015; Wang et al., 2018). On the other hand, astrocytes can also act protectively, as the rapid interferon (IFN) response after flavivirus infection restricts viral replication and spread through upregulating type I IFNs. This consequently enhances the expression of proteins that inhibit several steps of the host cell viral cycle, thus alleviating neuropathogenesis in the CNS (Schoggins and Rice, 2011; Lindqvist et al., 2016). These effects may influence region-specific multiple signaling mechanisms, as TBEV-, WNV-, and JEV-infected astrocytes and neurons showed better survival in certain regions of the brain (Hussmann et al., 2013; Lindqvist et al., 2016, 2018; Daniels et al., 2017).

### Flavivirus Strain-Related Effects on Astrocytes

#### WNV Strains

Interestingly, different flavivirus strains appear to exert different effects on specific astrocyte responses. This is perhaps most evident in the case of the WNV. On the one hand, several WNV strains were shown to be non-neuroinvasive (i.e., incapable of infecting the nervous system, especially the CNS) or poorly neuroinvasive, as demonstrated by intraperitoneal inoculation in mice and hamsters (Beasley et al., 2002). On the other hand, practically all WNV strains were shown to be neurovirulent (i.e., capable of causing disease of the CNS), as demonstrated by intracerebral inoculation in mice. Of particular note, WNV strains with longer passage histories were more virulent (i.e., resulted in decreased survival times of infected animals), as demonstrated by intranasal inoculation (Beasley et al., 2002). WNV favors entering the CNS via the hematogenous route, i.e., by crossing the BBB. As astrocytes play a key role in maintaining the functional integrity of the BBB, it is not surprising that they are considered important determinants of WNV infection. Hussmann et al. (2013) have shown that the replication of the avirulent strain WNV-MAD78 was delayed and reduced compared to the replication of the highly virulent strain WNV-NY in astrocytes but not in neurons or endothelial cells. This confirms that astrocytes play a critical role in WNV neuropathology. Mirroring the virulence of respective WNV strains, significantly higher levels of IFN (especially IFN-β) were detected in WNV-NY-infected compared to WNV-MAD78-infected human brain cortical astrocytes (Hussmann et al., 2013). Upon WNV infection, astrocytes also release and activate pro-inflammatory cytokines that recruit leukocytes and matrix metalloproteases, which disrupt the BBB (Van Marle et al., 2007; Verma et al., 2010, 2011). Such a strategy is not uncommon for neuroinvasive viruses (Eugenin et al., 2011; Růžek et al., 2011; Spindler and Hsu, 2012), as it enables an easier BBB breach for the second wave of viruses or even opens a new route for viral infections that utilized an alternative route in the first wave.

To date, three vaccines for WNV have been developed and licensed for equine use in Europe (Lecollinet et al., 2019); however, no vaccines have been licensed for human use to date. Animal experiments have confirmed that a vaccine derived from a chimeric virus, which was constructed using the structural proteins (M and E) of the Kunjin WNV strain and the genome backbone of the insect-specific Binjari virus, offered efficient protection against virulent WNV strains, including the highly pathogenic WNV_NY__99_ strain (Vet et al., 2020). In the WNV, the structural proteins E and M are responsible for binding to receptors, e.g., the dendritic cell- and liver/lymph node-specific intercellular adhesion molecule-3-grabbing non-integrins (DC-SIGN and L-SIGN, respectively), and have been associated with flavivirus-induced pathogenesis (Khoo et al., 2008; Basset et al., 2020). Hence, their function in the WNV is to some extent similar to the function of the spike (S) protein of SARS-CoV-2 (Huang et al., 2020). The emerging SARS-CoV-2 variant, in which the D614G mutation affects the spike (S) protein of SARS-CoV-2 strains from southern Europe, has rapidly spread and become the most prevalent genotype worldwide (Korber et al., 2020). The SARS-CoV-2 G614 variant replicated to higher titers in nasal-wash samples early after infection and outcompeted the D614 variant; however, the G614 variant did not cause more severe symptoms than the D614 variant in hamsters, which corroborates current findings in humans (Baric, 2020; Plante et al., 2020). Fortunately, the D614G substitution mutation is unlikely to reduce the ability of the vaccines in clinical trials to protect against COVID-19 (Plante et al., 2020).

#### ZIKV Strains

**FIGURE 3:**
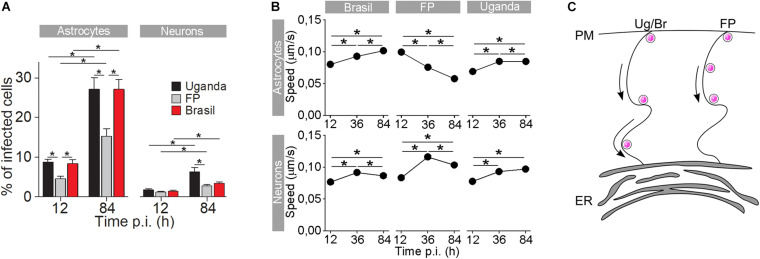
The rate of cell infection with Zika virus (ZIKV) and the speed of ZIKV-containing endocytotic vesicles depend on the ZIKV strain. **(A)** The percentages of ZIKV-positive astrocytes and neurons, infected with three different ZIKV strains [Brazil 2016 (Brazil), French Polynesia 2013 (FP), and Uganda #976 1947 (Uganda)] at 12 h post-infection (p.i.) and 84 h p.i. (means ± SEM; one-way ANOVA, *p < 0.05). The percentages were determined by counting the number of immunolabeled cells versus the number of all DAPI-stained nuclei (representing single cells). Data were collected from one experiment performed in duplicate. Results are based on a total of 10^4^ cells/group, counted in 16 independent fields of view (the numbers of cells counted per strain: 858–1805 astrocytes and 4882–5765 neurons). The percentages of ZIKV-positive astrocytes and neurons are different at both 12 h p.i. and 84 h p.i. in both cell types and increase with time. Note that the rate of cell infection depends on the ZIKV strain. **(B)** In astrocytes, the average speed of endocytotic vesicles increased with longer times p.i. for ZIKV-Br- and ZIKV-Ug-laden vesicles, while the average speed of ZIKV-FP-laden vesicles decreased. In neurons, the average vesicle speed exhibited the most prominent increase at 36 h p.i., regardless of the virus strain, and then declined the most in ZIKV-FP-laden vesicles. **(C)** A schematic representation of the higher speed of ZIKV-Br- and ZIKV-Ug-laden vesicles, as compared to the speed of ZIKV-FP-laden vesicles. The ZIKV is depicted as pink dots. Modified from Jorgačevski et al. (2019) with permission.

Similarly as with the WNV, a few amino acid alterations are also sufficient to notably change the neurovirulence, infection rate, and replication kinetics of the ZIKV (Duggal et al., 2017) as well as the differential survival of neural cell types (Retallack et al., 2016; Simonin et al., 2016; Hamel et al., 2017; Kuivanen et al., 2017; Jorgačevski et al., 2019). Higher infection rates in astrocytes compared to neurons can be attributed, in part, to the markedly different kinetics of the immune response triggered by different ZIKV strains (Hamel et al., 2017) but also to the expression levels of ZIKV receptors, including the tyrosine-protein kinase receptor Axl, a type of Tyro3, DC-SIGN, and T cell immunoglobulin mucin domain-1 (Lee et al., 2018), in contrast to neural progenitor cells and neurons in which Axl is poorly expressed (Nowakowski et al., 2016). Infection of human astrocytes with different ZIKV strains apparently leads to activation of the autophagy pathway (Ojha et al., 2019); however, the role of autophagy in ZIKV infection awaits further clarification. In addition, different ZIKV strains have been shown to differentially affect the traffic of endocytotic vesicles containing ZIKV (Figure 3; Jorgačevski et al., 2019). Specifically, the ZIKV strains from Uganda (Ug) and Brazil (Br) had markedly higher infection rates than the strain from French Polynesia (FP). Furthermore, endocytotic vesicles containing ZIKV Ug and ZIKV Br moved faster than those containing ZIKV FP (Jorgačevski et al., 2019). Moreover, the Asian ZIKV strain from Cambodia is more sensitive to the IFN-induced antiviral response than ZIKV Ug and ZIKV Br (Esser-Nobis et al., 2019).

A potential phosphomimetic 14-3-3-binding motif, termed 64-RLDP-67, is encoded in the non-structural protein 3 (NS3) of the three ZIKV strains studied (Ug, FP, and Br). This motif is identical to the motif found in WNV NS3, similar to the motif in dengue virus, and different from the motifs in yellow fever virus, hepatitis C virus, JEV, and TBEV (Riedl et al., 2019). The 14-3-3 proteins regulate numerous intracellular processes, including immunity (Tzivion et al., 2001; Nathan and Lal, 2020). ZIKV NS3 targets RIG-I-like-receptor-trafficking proteins of the 14-3-3 family to perturb innate immune signal transduction (Riedl et al., 2019). In the case of SARS-CoV-2, the ORF3a protein shares a high level (∼98%) of sequence similarity to the NS3 of bat CoV RaTG13 (Issa et al., 2020). The possible targeting of 14-3-3 proteins by ORF3a has not been fully addressed yet; however, ORF3a mutations may affect intraviral protein–protein interactions (Wu et al., 2020) and may cause higher COVID-19 mortality rates (Majumdar and Niyogi, 2020). As for the WNV, DC-SIGN/L-SIGN receptors are essential for optimal entry and infection by various ZIKV strains, and the glycan-binding domains and glycosylation of ZIKV E protein might play a role in the viral pathogenesis of different strains (Fernando et al., 2016; Carbaugh et al., 2019; Routhu et al., 2019).

#### TBEV Strains

The symptoms of TBEV infection, which may range from subclinical to mild flu-like disease to sever encephalitis with a lethal outcome, may depend on the virulence of the specific strain as well as the immune status of the host (Lindqvist et al., 2020). Similar to other flaviviruses, also the European TBEV subtype (TBEV-Eu), which is one of the three known TBEV subtypes, exhibits pronounced genetic variability with a considerable number of strains (Ecker et al., 1999; Fajs et al., 2012). Several TBEV strains have been documented to infect astrocytes, such as Ljubljana I, Neudoerfl, 93/783, Torö, and Hypr (Palus et al., 2014; Potokar et al., 2014; Fares et al., 2020; Lindqvist et al., 2020).

Comparing these strains, isolated from rodents, ticks, and humans, have shown that they differ, among others, in certain amino acids of envelope E protein, which is a cell attachment protein that enables cell entry. The E protein mediates the primary attachment of the virus to its target cell and mediates membrane fusion, thus determining, at least in part, the host-cell tropism and pathogenesis of the virus (Mandl et al., 2000; Kellman et al., 2018). For example, among the 15 amino acids that differ between the Ljubljana I and Neudoerfl strains, the most prominent amino acid change was identified to be I167V (Fajs et al., 2012). Both strains exerted a similar effect on cell viability. Primary rodent cortical astrocytes exhibited extraordinary resistance to the Ljubljana I strain in terms of uncompromised cell viability after 2 weeks of infection (Potokar et al., 2014). Similarly, the Neudoerfl strain, a prototype strain of the European subtype, also did not extensively compromise the viability of primary human brain cortical astrocytes after 2 weeks of infection (Palus et al., 2014). Unfortunately, the two strains were not directly compared in the same astrocyte culture in terms of their replication and effect on cell viability. However, a clear difference in neurovirulent properties was documented between the 93/783 and Torö strains (Lindqvist et al., 2020). These two strains also exhibit differences in their E protein properties, which result in enhanced binding and host cell entry of the more virulent strain 93/783. These effects were evident in neurons, while in astrocytes, no differences were noted between both strains in their host cell entry efficacy, but only in their replication rate. The latter was far higher in the 93/783 strain with the A83T and A463S amino acid substitutions, which are not located on E protein (Lindqvist et al., 2020). Differences between various strains were also noted at the level of mice survival, as 93/783-infected mice showed shorter survival times than Torö-infected mice. Additionally, 93/783 was more neuroinvasive than Torö, as higher quantities of 93/783 were located in the olfactory bulb, cerebrum, and brain stem, which also correlated with higher leukocyte common antigen CD45 RNA levels following 93/783 infection (Lindqvist et al., 2020). Although the afore-mentioned differences cannot be attributed solely to changes in the E protein, it has been shown that the E protein of 93/783 exacerbated the clinical outcome, pathogenicity, and neurovirulence of TBEV. All of these changes correlated with increased levels of viral RNA and infiltrating T and B cells in the brain (Lindqvist et al., 2020). In addition, E protein variations between strains influence the formation of neutralizing antibodies against E protein that are important in neutralizing the virus after infection. As amino acid differences in the E protein are known to affect neutralizing antibodies, vaccine-derived antibodies are not equally efficient for different strains of TBEV and thus these E protein variations might be a contributing factor to vaccine breakthroughs (Beck et al., 2016; Lindqvist et al., 2020).

Different TBEV strains also show high longevity in host organs; however, the amount of viral load in particular organs appears to differ between host organisms and respective strains. For example, when comparing the strains Ljubljana I and Hypr, the presence of TBEV RNA was confirmed for a longer period of time after the detection of TBEV-specific antibodies. In Ljubljana I-infected forest rodents *Myodes glareolus* and *Apodemus sylvaticus*, the highest viral load was measured in spleen and brain samples (Knap et al., 2012). These viral loads were much higher in Hypr strain RNA-infected laboratory-bred *Microtus arvalis*, in which RNA was confirmed in several organs even several months after infection (Achazi et al., 2011). Mutations that increase the net positive charge of the E protein lead to lower virus pathogenicity and neuroinvasiveness due to stronger binding to glycosaminoglycans and thus a more rapid clearance of the virus from the blood, as was demonstrated in mice infected with Far-Eastern subtype Oshma strains (Goto et al., 2003). Genetic divergence of strains that circulate in the population from the vaccine strain might reduce neutralizing antibody titers and thus decrease vaccine efficacy (Lindqvist et al., 2020).

## Conclusion

While the pathophysiology of the neurotropic mechanisms of the new SARS-CoV-2 are yet to be confirmed, it is likely, based on the mechanisms known for other neurotropic viruses, including TBEV, WNV, and ZIKV, that astrocytes play a key role in the pathophysiology of COVID-19. Astrocytes may as such be crucially involved in SARS-CoV-2 production and spread in the CNS, immunomodulatory responses, and survival of neurons. These processes may be diversely affected in different parts of the brain that express variable amounts of SARS-CoV-2 receptors. In analogy with neurotropic flavivirus strains, it is expected that different SARS-CoV-2 strains may trigger somewhat different neurological symptoms as well as variations in the response to different vaccines.

## Author Contributions

All the authors wrote the manuscript, extensively contributed to the preparation and finalization of the manuscript, and approved the manuscript for publication.

## Conflict of Interest

The authors declare that the research was conducted in the absence of any commercial or financial relationships that could be construed as a potential conflict of interest.
